# Six Types of Tea Reduce Acute Alcoholism in Mice by Enhancing Ethanol Metabolism, Suppressing Oxidative Stress and Inflammation

**DOI:** 10.3389/fnut.2022.848918

**Published:** 2022-05-23

**Authors:** Xingfei Lai, Xinrong Wang, Shuai Wen, Lingli Sun, Ruohong Chen, Zhenbiao Zhang, Qiuhua Li, Junxi Cao, Zhaoxiang Lai, Zhigang Li, Shili Sun, Xiaohui Liu

**Affiliations:** ^1^College of Tea Science, Yunnan Agricultural University, Kunming, China; ^2^Guangdong Provincial Key Laboratory of Tea Plant Resources Innovation and Utilization, Tea Research Institute, Guangdong Academy of Agricultural Sciences, Guangzhou, China

**Keywords:** *Camellia sinensis*, tea, acute alcoholism, alcoholic liver injury, ethanol metabolism, oxidative stress, inflammation

## Abstract

Acute alcoholic intoxication (AAI) is a pathological process of multiple system damage caused by a large amount of alcohol, especially in the liver. Although tea extracts alleviate AAI and alcohol-induced liver damage, the mechanisms underlying the protective actions of different types of Chinese tea are unclear. In this study, the AAI mice model was used to explore the functions and mechanisms of six types of tea extract (WEATs) in alleviating AAI. The losing righting reflexes of mice were evaluated to assess the effects of the WEATs on AAI. The levels of the ethanol metabolism enzymes (ADH, ALDH2, CYP2E1), the oxidative stress-related indicators (NRF-2, HO-1, SOD, GSH, CAT, and TG) and the inflammatory factors (TNF-α, iNOS, IL-6, and IL-10) were determined. Black tea and dark tea significantly shortened the sleep time (duration of the loss of righting reflex) and had a good sobering effect. Green tea and oolong tea had the dual effect of prolonging tolerance time (time of losing righting reflex) and shortening sleep time. While white tea had the most significant effect on prolonging tolerance time but with no obvious sobering effect. Black tea, dark tea, and oolong tea significantly up-regulated ADH and ALDH2, and down-regulated CYP2E1. Green tea and white tea significantly increased the levels of Nrf2, GSH, and CAT. Black tea, dark tea and oolong tea markedly increased the levels of HO-1, IL-10, and inhibited TG. Therefore, it is possible that black tea, dark tea and oolong tea reduced AAI by increasing ethanol metabolism, suppressing oxidative stress and inflammation. While green tea was mainly by regulating oxidative stress. White tea may prolong the tolerance time by increasing ethanol metabolism and reducing oxidative stress. Different types of tea have specific chemical compositions and can alleviate AAI. In conclusion, despite variations in the composition and mechanism of action, tea is a potent natural product to alleviate a hangover and protect the liver.

## Introduction

Acute alcoholic intoxication (AAI), commonly known as drunkenness is a pathological process that damages the liver, heart, kidneys, and other organs caused by a large amount of alcohol ingested in a short period of time (1). Since the absorption is greater than the metabolism of alcohol, the absorbed alcohol enters the brain through the blood-brain barrier and inhibits the excitability of the central nervous system. In severe cases, it will endanger the cardiovascular system, digestive system, immune system, nervous system and even lead to death (2–4). Alcohol is responsible for approximately 4% of all deaths annually and 5% of all disabilities worldwide, owing to a large number of alcohol-associated diseases as well as injuries caused by traffic accidents and violence (5). Additionally, the harmful effects of alcohol particularly affect those of working age, with 139 million disability-adjusted life years lost, or 5.1% of the total global burden of disease, attributable to alcohol consumption (6). Although anti-alcoholic drugs including disulfiram (7), naltrexone and acamprosate (8) have been reported to have the anti-alcohol effect, there are few drugs currently available to treat humans. Therefore, the development of safe and effective natural products for alleviating AAI is of interest globally.

Tea is a major beverage consumed worldwide and has various health benefits. In China, tea is classified into six categories according to the degree of fermentation, including green tea (non-fermented tea), yellow tea (micro-fermented tea), white tea (lightly fermented tea), oolong tea (semi-fermented tea), black tea (fully fermented), and dark tea (post-fermented tea) (9). Several studies indicate that tea has a protective effect against acute alcoholism and alleviates ethanol-induced liver injury, and also exerts very strong antioxidant and anti-inflammatory activities *in vivo* (10–13). Although there have been several studies reporting the anti-alcoholism activity of different kinds of tea, most of them focus on a single or a few categories, or a single active constituent such as (−) Epigallocatechingallate (EGCG) (14, 15), theaflavins (16) or L-theanine (17). Due to the differences in the degree of fermentation, the six major teas have different biochemical compositions and may have various protective effects on acute alcoholism. Therefore, it is very meaningful to systematic evaluate the anti-alcoholism activity of six categories of tea.

In this study, the six types of tea were all made from the same batch of Yinghong No. 9 fresh leaves and used to explore the functions and mechanisms in alleviating AAI *in vivo*. In addition, a correlation analysis was conducted on the content of active components of the six types of tea and acute alcoholism-related Indicators. The results revealed the differences of effects and mechanisms among the six types of tea in alleviating AAI, and providing new ideas for developing natural products to relieve alcoholism and protect the liver.

## Materials and Methods

### Preparation of Tea Water Extracts

The six types of tea were processed from the Yinghong No. 9 tea tree (*Camellia sinensis var. assamica cv. Yinghong 9*) planted by the Tea Research Institute of the Guangdong Academy of Agricultural Sciences in Yinghong town, Yingde city, Guangdong province. Yinghong No. 9 is an excellent clonal tea variety and suitable for producing into different kinds of tea. Besides, it has been widely naturalized in Guangdong province owning to its high quality and high economic value. The six types of tea used in this study were all made from the same batch of Yinghong No. 9 fresh leaves with one bud and two leaves in May 2020. For green tea, the fresh leaves were processed at 280°C to inactivate the enzymes, kneaded for 45 min, and then dried, whereas the white tea was prepared by drying the fresh leaves after 48 h of indoor withering. Yellow tea was prepared by withering the fresh leaves in the room for 6 h, then heating to 180°C to inactivate most enzymes before kneading for 45 min, and then the tea leaves were yellowed at room temperature for 12 h and dried. Oolong tea was processed by wilting the fresh leaves in the sun for 5 min, withering in the room for 3 h, shaking every hour five times. After quenching at 280°C, the leaves were twisted for 45 min and dried. Black tea was prepared by withering the fresh leaves indoors for 12 h, kneading for 45 min, fermenting for 8 h, and dried. Dark tea was processed by heating the fresh leaves to 180°C to inactivate the enzymes, then kneaded for 45 min and dried in the sun. The teas were prepared by making up 500 kg of dried tea leaves with 5% water, piled for 40 days, and dried. The water extracts (WEATs) were prepared as follows: after the tea was crushed, it was extracted for 20 min in water (tea/water (w/v) 1:20), suction filtered, and extracted once more. After the tea filtrate was combined, the filtrate was concentrated in a vacuum rotary evaporator. The concentrated solution was lyophilized into a powder using a freeze dryer, dried, and stored for later use.

### Determination of the Biochemical Components of Tea

The folin-phenol method was used to measure the tea polyphenols (18) and the anthronecolorimetry was used to measure the total soluble sugar content (19). Free amino acids were quantified by the ninhydrin method and flavonoids were determined by AlCl_3_ colorimetry (20). The content of catechins, caffeine, gallic acid, and L-theanine were measured by high-performance liquid chromatography (HPLC) (21, 22).

### Establishment of an Acute Alcoholic Intoxication Mice Model

Six-week-old male C57BL/6J mice (20 ± 2 g) were obtained from the Beijing Vital River Laboratory Animal Technology Co., Ltd., China. All experimental procedures were conducted per the institutional guidelines for the care and use of laboratory animals, and all efforts were made to minimize animal suffering. The protocols were approved by the Ethical Committee of the Tea Research Institute (2016-002), Guangdong Academy of Agricultural Sciences. All mice were kept under controlled temperature (22 ± 1°C) and humidity (60 ± 15%) in a 12 h light/dark cycle with free access to deionized water and basic food. After 1 week of acclimatization, forty male C57BL/6J mice were randomly divided into four groups and fasted for 12 h but with access to water. Then, the mice were gavaged with 56° liquor in the doses of 0.10, 0.20, and 0.30 ml/kg⋅BW. The normal group was given the same volume of distilled water. After 30 min, the losing righting reflex was observed and recorded to determine the optimal dose to induce AAI, which was 0.20 ml/kg⋅BW. The loss of the righting reflex was judged present if the test animals could not right themselves within 30 s to the prone position with all four paws.

### Assessment of the Drunken and Sober Time

Eighty male C57BL/6J mice were randomly divided into eight groups, namely the control group (CON), model group (MOD), and WEATs groups (including green tea (GRE), white tea (WHI), yellow tea (YEL), oolong tea (OOL), black tea (BLA), and dark tea (DAR) groups). Before the experiment, the mice were fasted for 12 h with access to water. The WEATs groups were administered intragastrically in one dose (1,000 mg/kg⋅BW) and the normal and model groups received the same volume of distilled water. One hour after administration, all groups except the normal group were given intragastrically 56° liquor at a dose of 0.20 ml/kg⋅BW. The drunken and sober times of the mice were recorded.

### Detection of Markers in the Liver and Serum

An additional 80 male C57BL/6J mice were randomly divided into eight groups and treated with WEATs as described above. After intragastric administration of liquor for 30 min, the mice were anesthetized and sacrificed to harvest the serum and livers.

### Liver Index Determination

The mice before sacrifice and the harvested livers were weighed and the organ index calculated as follows:


Organindex(%)=(organmass/weight)×100%

### Determination of Aminotransferase, Aminotransferase, Superoxide Dismutase, Glutathione, Catalase, and Triglyceride

The activity or content of alanine aminotransferase (ALT), aminotransferase (AST), superoxide dismutase (SOD), glutathione (GSH), catalase (CAT), and triglyceride (TG) in the serum and liver were determined using kits from Nanjing Jiancheng Bioengineering Institute, China according to the manufacturer’s instructions.

### Determination of Alcohol Dehydrogenase and Aldehyde Dehydrogenase

ELISA kits (Beijing Solarbio Science and Technology Co., Ltd.) were used to determine the activity of alcohol dehydrogenase (ADH) and aldehyde dehydrogenase (ALDH) in the serum and liver per the manufacturer’s instructions.

### Histological Examination

The formalin-fixed liver was dehydrated in an ethanol gradient and embedded in paraffin. Sections of 4 μm thickness were cut, deparaffinized, rehydrated, stained with hematoxylin and eosin (H&E), then examined under a light microscope (Olympus, Tokyo, Japan). The area of each adipocyte was quantified using ImageJ software (National Institutes of Health, Bethesda, MD).

### Western Blotting

Proteins were extracted from the liver tissues using a lysis buffer at 4°C. The extracts were centrifuged at 18,000 g at 4°C for 30 min and the protein content of the supernatants was determined. Equal amounts of protein per sample were separated by 10% SDS-PAGE and transferred to PVDF membranes, which were blocked at room temperature with 5% skim milk in TBST for 1 h, then incubated overnight with primary antibodies against ADH1A/ADH1B/ADH1G, ALDH2 (Boster Biological Technology Co., Ltd., Beijing, China), iNOS, NRF2, HO-1(Cell Signaling Technology, Inc., United States), IL-6, CYP2E1 TNF-α, IL-10 (Abcam) and β-actin (Cell Signaling Technology, Inc., United States) at 4°C. After washing thrice with TBST, the blots were incubated with secondary antibodies conjugated to horseradish peroxidase. The protein bands were visualized by enhanced chemiluminescence.

### Immunohistochemistry

Tissue sections were dewaxed and rehydrated, then sequentially soaked in water for 2 min, TBS (Beijing Solarbio Science and Technology Co., Ltd.) for 5 min, and 3% H_2_O_2_ for 15 min in the dark to quench endogenous peroxidases. The sections were rinsed with tap water, soaked in TBS for 5 min, and microwaved in EDTA solution for antigen retrieval. After cooling to room temperature, the sections were washed thrice with TBS, demarcated with an oil pen, and blocked with 5% goat serum (Boster Biological Technology Co., Ltd., Beijing, China) for 30 min at room temperature. The blocking buffer was gently blotted out and the sections were incubated overnight with suitable primary antibodies at 4°C in a wet box. After washing thrice with TBS, the sections were incubated with secondary antibodies (Boster, Wuhan, China) at room temperature for 1 h and washed with TBS. The sections were then incubated with HRP-labeled streptavidin (Beyotime, Shanghai, China) for 1 h at room temperature and washed thrice with TBS, and the color was developed using DAB reagent. After rinsing with tap water, the sections were counterstained with hematoxylin (Beyotime, Shanghai, China) for 1 min, and held under running tap water until all the excess dye was washed out. Finally, the sections were dehydrated and sealed using neutral balsam (Beijing Solarbio Science and Technology Co., Ltd.) and observed under an Olympus BX-53 microscope.

### Statistical Analysis

All statistical analyses were conducted using Prism 8.0 software for Windows (GraphPad Software, La Jolla, CA, United States). Multiple groups were compared by analysis of variance (ANOVA). *P*-values < 0.05 were considered statistically significant. Data are presented as the mean ± SEM.

## Results

### Composition of the Six Types of Tea

Table 1 shows the ingredients and content of WEATs. Among the six types of tea, green tea had the highest content of tea polyphenols (23.33 ± 0.09%), followed by yellow tea and oolong tea. Dark tea had the lowest tea polyphenol content (7.22 ± 0.31%). The content of free amino acids and L-theanine was highest in non-fermented tea (green tea and yellow tea), followed by lightly fermented tea (white tea and oolong tea) and fully fermented tea (black tea and dark tea). The content of soluble sugar was highest in green tea (9.31 ± 0.04%) and lowest in black tea (4.90 ± 0.02%). The content of flavonoids was highest in black tea (17.06 ± 0.15 mg/kg) and lowest in black tea (7.26 ± 0.19 mg/kg). The content of caffeine was the highest in black tea (48.31 ± 0.29 mg/kg) and lowest in oolong tea (30.13 ± 0.66 mg/kg). The content of gallic acid was the highest in dark tea (2.14 mg/g), followed by oolong tea (1.96 mg/g), and green tea (0.12 mg/g). The content of catechins C, EC, EGCG, GCG, and ECG was highest in non-fermented tea (green tea and yellow tea), followed by lightly fermented tea (white tea and oolong tea), and fully fermented tea (black tea and dark tea).

**TABLE 1:** Analysis of the active components in six kinds of tea.

Constituent	Green tea	White tea	Yellow tea	Oolong tea	Black tea	Dark tea
Tea polyphenols (%)	23.33 ± 0.09	15.89 ± 0.37	22.34 ± 0.20	17.38 ± 0.21	10.71 ± 0.41	7.22 ± 0.31
Free amino acid (%)	4.19 ± 0.05	2.80 ± 0.01	4.48 ± 0.08	3.25 ± 0.01	2.39 ± 0.01	1.22 ± 0.01
L-theanine (%)	2.30 ± 0.01	1.21 ± 0.01	1.94 ± 0.06	1.64 ± 0.02	1.29 ± 0.02	0.22 ± 0.00
Soluble sugar (%)	9.31 ± 0.04	5.86 ± 0.07	8.95 ± 0.14	5.23 ± 0.05	4.90 ± 0.02	5.48 ± 0.18
Flavonoids (mg/g)	8.24 ± 0.04	10.88 ± 0.02	8.50 ± 0.16	8.74 ± 0.17	17.06 ± 0.15	7.26 ± 0.19
Caffeine (mg/g)	39.82 ± 0.40	33.53 ± 1.10	38.33 ± 0.26	30.13 ± 0.66	36.27 ± 0.75	48.31 ± 0.29
Gallic acid (mg/g)	0.09 ± 0.03	1.12 ± 0.07	1.06 ± 0.08	1.96 ± 0.04	1.23 ± 0.06	2.14 ± 0.01
GC (mg/g)	13.87 ± 0.03	72.02 ± 1.25	11.15 ± 0.13	6.51 ± 0.10	61.90 ± 1.47	111.70 ± 0.33
EGC (mg/g)	36.89 ± 0.70	8.06 ± 0.31	31.01 ± 0.55	16.54 ± 0.31	9.40 ± 0.20	−
C (mg/g)	14.07 ± 0.12	4.09 ± 0.19	13.37 ± 0.07	5.95 ± 0.20	1.46 ± 0.06	0.79 ± 0.01
EC (mg/g)	20.25 ± 0.11	4.19 ± 0.22	18.72 ± 0.04	9.62 ± 0.24	0.90 ± 0.02	1.07 ± 0.02
EGCG (mg/g)	66.59 ± 0.56	19.54 ± 0.50	53.29 ± 0.47	26.62 ± 0.17	4.85 ± 0.07	0.76 ± 0.00
GCG (mg/g)	4.02 ± 0.10	1.61 ± 0.03	3.29 ± 0.05	1.88 ± 0.00	0.98 ± 0.04	−
ECG (mg/g)	48.82 ± 0.19	28.92 ± 0.73	46.30 ± 0.66	24.01 ± 0.19	5.53 ± 0.05	0.45 ± 0.06
CG (mg/g)	0.24 ± 0.03	0.48 ± 0.02	1.76 ± 0.01	0.87 ± 0.02	0.63 ± 0.03	0.28 ± 0.02

*Data are presented as the mean ± SEM (n = 3).*

*GC, Gllocatechin; EGC, epigallocatechin; C, catechin; EC, epicatechin; EGCG, epigallocatechin-3-gallate; GCG, gallocatechin-3-gallate; ECG, epicatechin-3-gallate; and CG, catechingallate. “−” represents the value was below the detection limit.*

### Effects of Water Extracts on Losing Righting Reflex

**FIGURE 1:**
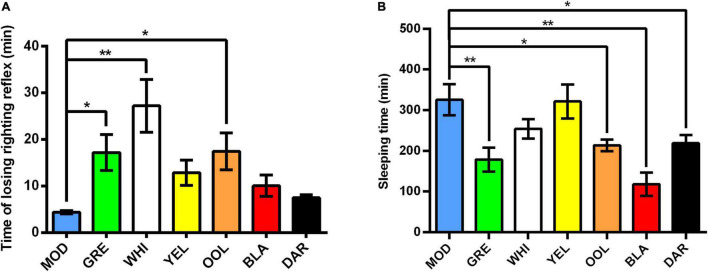
Effect of WEATs treatment on the time of losing righting reflex **(A)** and sleeping time **(B)**. Each value represents the mean ± SEM (*n* = 10). *p < 0.05 vs. MOD; **p < 0.01 vs. MOD.

As shown in Figure 1A, compared with the model group, the tolerance time (time of losing righting reflex) of mice treated with WEATs was prolonged, significantly for white tea (*p* < 0.01) as well as green tea and oolong tea (*p* < 0.05). The results in Figure 1B showed that the sleep time (the duration of the loss of righting reflex) of mice treated with WEATs was shortened to varying degrees compared with the model group. Black tea (*p* < 0.01) were most effective followed by green tea (*p* < 0.01), dark tea (*p* < 0.05), and oolong tea (*p* < 0.05).

### Water Extracts Regulated the Levels of Alcohol Dehydrogenase, Aldehyde Dehydrogenase, Aminotransferase, and Aminotransferase in Serum

**FIGURE 2:**
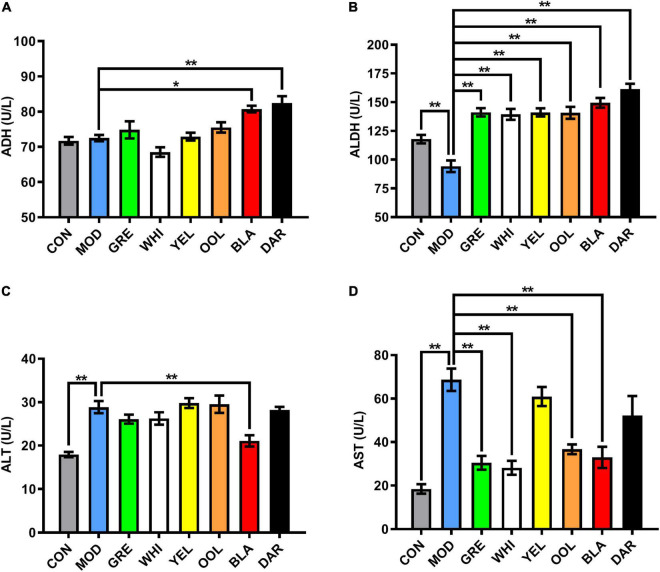
Effect of WEATs treatment on ADH **(A)**, ALDH **(B)**, ALT **(C)** and AST **(D)** levels in the serum. Each value represents the mean ± SEM (*n* = 10). *p < 0.05 vs. MOD; **p < 0.01 vs. MOD.

Compared with the normal group of mice, there was no significant difference in the activity of ADH in the serum of the model group, whereas ADH activity was significantly increased in the serum of mice administered dark tea (*p* < 0.01) and black tea (*p* < 0.05) (Figure 2A). The activity of ALDH in the serum of mice in the model group was significantly lower than that in the normal group (*p* < 0.01) and significantly increased in all WEATs treated groups (*p* < 0.01, Figure 2B). The activity of ALT in the serum of the model group was significantly higher than that of the normal group (*p* < 0.01) and decreased in the serum of mice in each WEATs group, with the serum ALT activity of the black tea group significantly lower than the model group (*p* < 0.01, Figure 2C). The activity of AST in the serum of the model group was significantly higher than that of the normal group (*p* < 0.01). Compared with the model group, the activity of AST was lower in the serum of mice administered with WEATs, with green tea, white tea, oolong tea, and black tea significantly reducing AST activity (*p* < 0.01, Figure 2D).

### Water Extracts Attenuated Liver Injury in the Acute Alcoholic Intoxication Mouse Model

**FIGURE 3:**
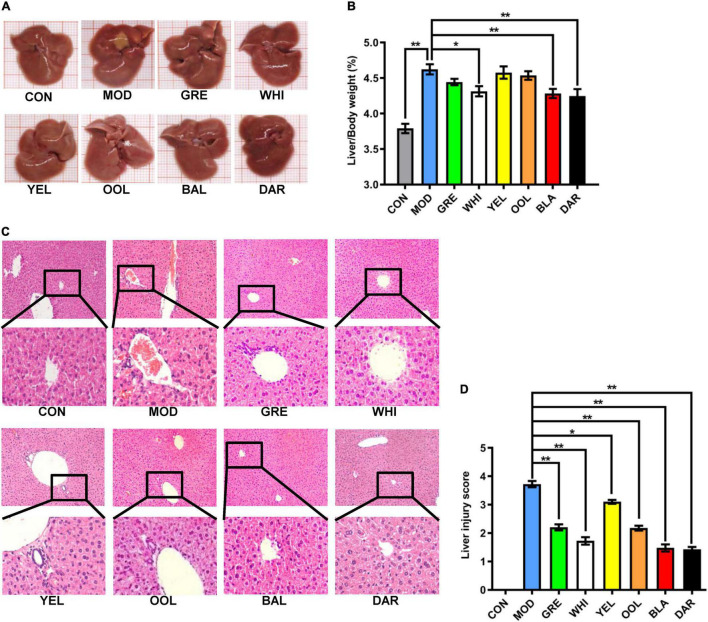
Effect of WEATs treatment on liver morphology **(A)**, liver/body index **(B)**, HE staining **(C)**, liver injury score **(D)**. Each value represents the mean ± SEM (*n* = 10). *p < 0.05 vs. MOD; **p < 0.01 vs. MOD.

The liver of mice in the normal group was reddish-brown, bright in color, and soft in texture, whereas the liver in the AAI model mice was dark red and some tissues had obvious lesions. However, the liver of mice treated with WEATs was reddish-brown and the color was brighter, with no significant liver lesions (Figure 3A). The liver index data was significantly higher in the model group compared to the normal group (*p* < 0.01) and significantly lower in the black tea, dark tea (*p* < 0.01), and white tea groups (*p* < 0.05) than the model group (Figure 3B). HE staining of the liver showed that the AAI model group had hepatocyte disorder, unclear cell boundaries, patchy lesion or cell necrosis, and moderately increased inflammatory cell infiltration. After WEATs treatment, hepatocytes, and hepatic cords were significantly improved, the liver cell cord was arranged neatly, with a small amount of inflammatory cell infiltration (Figure 3C). The liver injury score of mice in each WEATs treatment group was significantly lower than those of the model group (Figure 3D), indicating that the six types of tea effectively improved the liver damage caused by AAI.

### Water Extracts Regulated the Levels of Alcohol Dehydrogenase, Aldehyde Dehydrogenase, Aminotransferase, Aminotransferase, Superoxide Dismutase, Glutathione, Catalase, and Triglyceride in Mouse Liver

**FIGURE 4:**
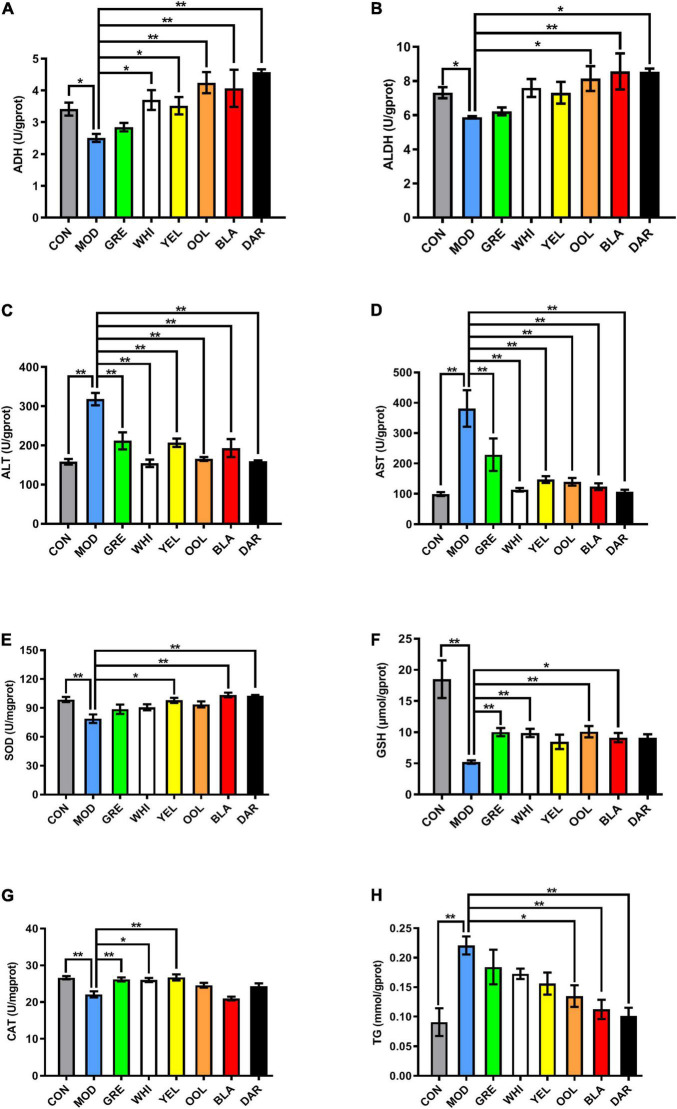
Effect of WEATs treatment on ADH **(A)**, ALDH **(B)**, ALT **(C)**, AST **(D)**, SOD **(E)**, GSH **(F)**, CAT **(G)**, TG **(H)** and levels in the liver. Each value represents the mean ± SEM (*n* = 10). *p < 0.05 vs. MOD; **p < 0.01 vs. MOD.

The activity of ADH (Figure 4A) and ALDH (Figure 4B) in the liver of model group mice decreased (*p* < 0.05), with oolong tea, black tea, dark tea (*p* < 0.01), yellow tea, and white tea (*p* < 0.05) significantly increasing ADH activity compared to the model group. Black tea (*p* < 0.01), oolong tea, and dark tea (*p* < 0.05) significantly increased ALDH activity. The activity of ALT (Figure 4C) and AST (Figure 4D) in the liver of the model group mice was increased (*p* < 0.01), while WEATs significantly reduced the activity of ALT and AST (*p* < 0.01). The activity of SOD (Figure 4E), GSH (Figure 4F), and CAT (Figure 4G) in the liver of the model group were lower compared to the normal group (*p* < 0.01), with black tea, dark tea (*p* < 0.01), and white tea (*p* < 0.05) significantly increasing the SOD level in the liver of AAI mice. Green tea, white tea, oolong tea (*p* < 0.01), and black tea (*p* < 0.05) significantly increased GSH levels. The CAT activity in the green tea group, yellow tea group (*p* < 0.01), and white tea group (*p* < 0.05) increased significantly. Regarding the activity of TG, the activity in the model group was higher than the normal group (*p* < 0.01), with black tea, dark tea (*p* < 0.01), and oolong tea (*p* < 0.05) significantly increasing TG activity (Figure 4H).

### Water Extracts Regulated ALDH2 and CYP2E1 Expression in the Liver of Acute Alcoholic Intoxication Model

**FIGURE 5:**
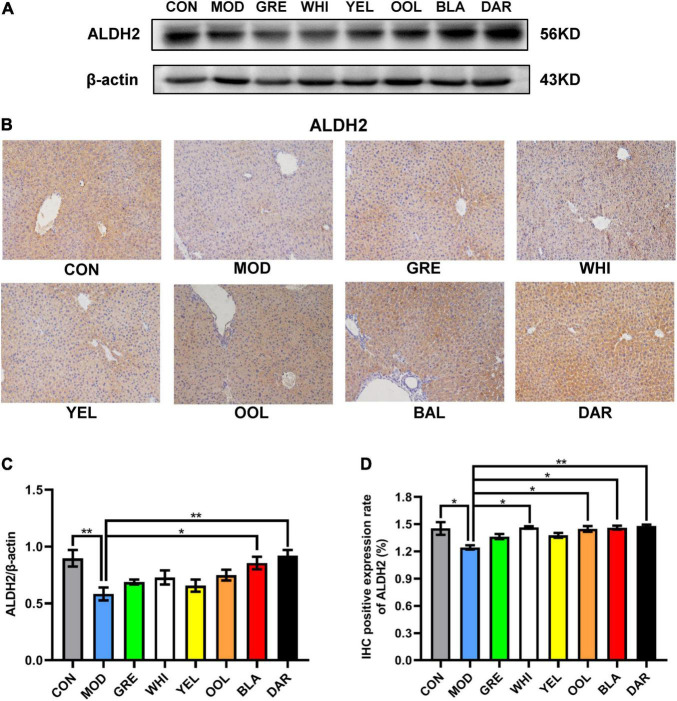
Effects of WEATs on ALDH2 expression in the liver of mice. Representative immunoblots **(A)** and IHC images **(B)** of ALDH2 expression in the liver. Quantification of ALDH2 expression by western blotting **(C)** and IHC **(D)**. Each value represents the mean ± SEM (*n* = 9). *p < 0.05 vs. MOD; **p < 0.01 vs. MOD.

ALDH2 is a key enzyme in ethanol metabolism, responsible for catalyzing the oxidation of acetaldehyde to acetic acid, which determines the speed of ethanol metabolism. The western blotting (Figure 5A) and immunohistochemistry (IHC) (Figure 5B) results showed that compared with the normal group, the expression of ALDH2 in the liver of the AAI model group was reduced, with black tea (*p* < 0.01) and dark tea (*p* < 0.05) promoting an increase in ALDH2 expression in the liver of AAI model mice (Figure 5C). Similarly, the immunohistochemical results showed that the expression of ALDH2 in the dark tea group (*p* < 0.01), white tea group, black tea group, and oolong tea group increased significantly (*p* < 0.05) (Figure 5D).

**FIGURE 6:**
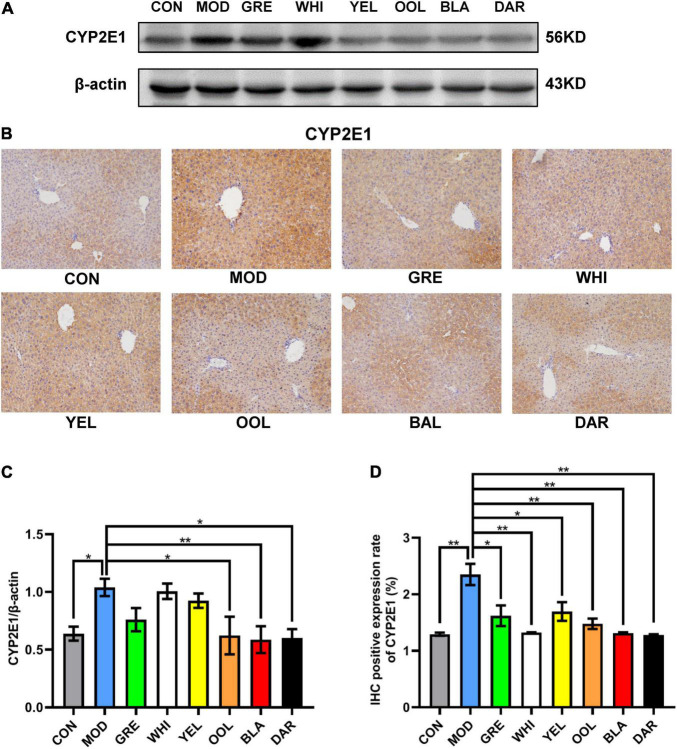
Effects of WEATs on CYP2E1 expression in the liver of mice. Representative immunoblots **(A)** and IHC images **(B)** of CYP2E1 expression in the liver. Quantification of CYP2E1 expression by western blotting **(C)** and IHC **(D)**. Each value represents the mean ± SEM (*n* = 10). *p < 0.05 vs. MOD; **p < 0.01 vs. MOD.

CYP2E1 is an effective active oxygen-generating enzyme, which produces superoxide anion free radicals and hydrogen peroxide to induce liver damage caused by ethanol. The results of western blotting (Figure 6A) and IHC (Figure 6B) showed that the expression of CYP2E1 in the liver of the model group was higher than in the normal group. Western blotting statistical data showed that black tea (*p* < 0.01), oolong tea, and dark tea (*p* < 0.05) significantly reduced the expression of CYP2E1 in the AAI model mice (Figure 6C). Immunohistochemical statistical data showed that white tea, oolong tea, black tea, dark tea (*p* < 0.01), green tea, and yellow tea (*p* < 0.05) all significantly reduced the expression of CYP2E1 (Figure 6D). These data prove the role of WEATs in regulating ethanol metabolism during AAI remission.

### Water Extracts Regulated TNF-α, iNOS, Nrf2, HO-1, IL-6, and IL-10 Expression in the Liver of the Acute Alcoholic Intoxication Model

**FIGURE 7:**
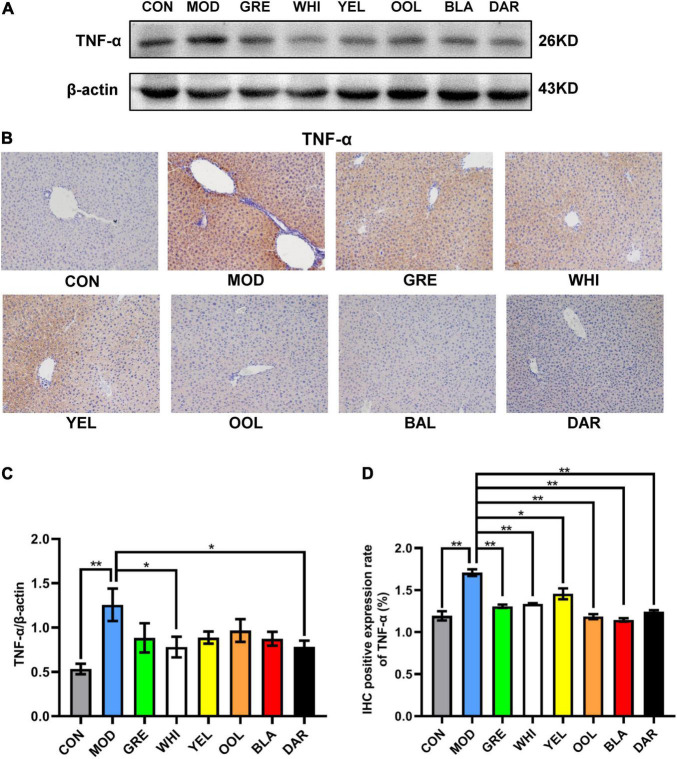
Effects of WEATs on TNF-α expression in the liver of mice. Representative immunoblots **(A)** and IHC images **(B)** of TNF-α expression in the liver. Quantification of TNF-α expression by western blotting **(C)** and IHC **(D)**. Each value represents the mean ± SEM (*n* = 10). *p < 0.05 vs. MOD; **p < 0.01 vs. MOD.

**FIGURE 8:**
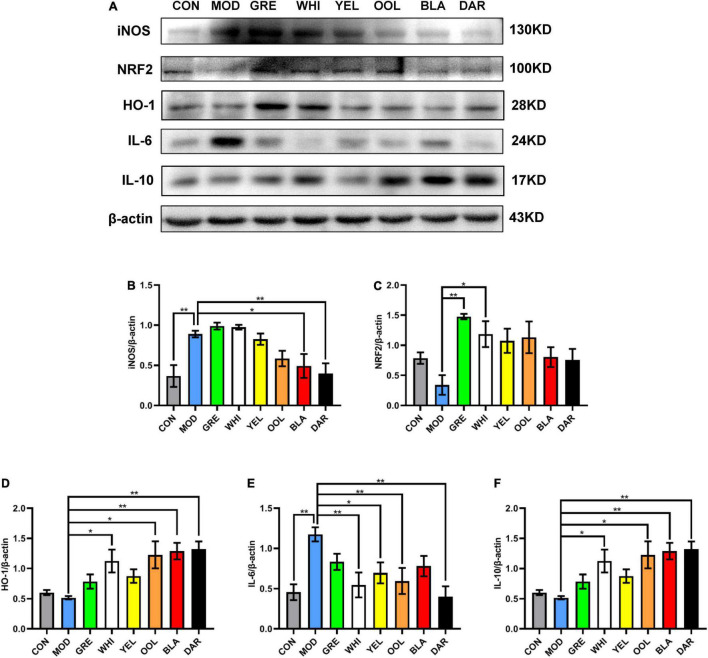
Effects of WEATs on iNOS, NRF2, HO-1, IL-6, and IL-10 expression in the liver of mice. Representative immunoblots **(A)** of iNOS, NRF2, HO-1, IL-6, and IL-10 expression in the liver. Quantification of iNOS **(B)**, NRF2 **(C)**, HO-1 **(D)**, IL-6 **(E),** and IL-10 **(F)** expression by western blotting. Each value represents the mean ± SEM (*n* = 10). *p < 0.05 vs. MOD; **p < 0.01 vs. MOD.

To explore the effects of six types of tea on the inflammatory response caused by acute alcoholism, we analyzed the expression of inflammatory factors such as TNF-α, iNOS, NRF2, HO-1, IL-6, and IL-10 in the liver of mice. Western blotting and immunohistochemical results showed that AAI promoted the expression of TNF-α in the liver (Figures 7A,B). The statistical results showed that WEATs reduced the expression of TNF-α in mice in the AAI model group (Figures 7C,D). Western blotting results showed that compared to the normal group, the expression of iNOS and IL-6 in the liver of the model group increased, and the expression of NRF2, HO-1, and IL-10 decreased (Figure 8A). Compared with the model group, except for the green tea group and the white tea group, the expression of iNOS in the other WEATs groups was reduced (Figure 8B). All WEATs increased the expression of NRF2, HO-1, and IL-10 in the liver of AAI mice (Figures 8C,D,F), and decreased the expression of IL-6 (Figure 8E), indicating that WEATs help to improve liver inflammation caused by AAI.

### Correlation Analysis of the Active Components of Six Types of Tea and Acute Alcoholism-Related Indicators

**FIGURE 9:**
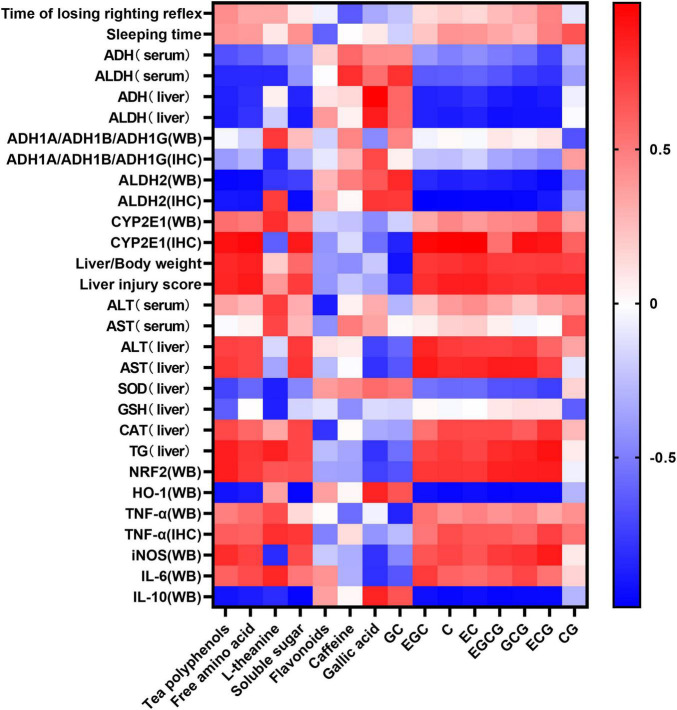
Heat map of composition of the six types of tea and related indicators of acute alcoholism. Blue and red represent negative correlation and positive correlation, respectively.

The statistical heatmap in Figure 9 shows that the components of tea polyphenols, free amino acid, L-theanine, soluble sugar, gallic acid, EGC, C, EC, EGCG, GCG, and ECG were positively correlated with the losing time of the righting reflex and sleep time in mice. The catechingallate (CG) level was negatively correlated with the losing time of the righting reflex in mice and positively correlated with sleep time, while the level of flavonoids, caffeine, and GC was negatively correlated with the losing time of the righting reflex and sleep time in mice. Tea polyphenols, free amino acid, L-theanine, soluble sugar, gallic acid, EGC, C, EC, EGCG, GCG, ECG, and CG were negatively correlated with the activity of ADH and ALDH2 in mouse serum and liver, and flavonoids, caffeine, and GC were positively correlated. Tea polyphenols, free amino acid, L-theanine, soluble sugar, gallic acid, EGC, C, EC, EGCG, GCG, ECG, and CG were negatively correlated with the protein expression of ALDH2 in mouse liver and positively correlated with CYP2E1, while the level of flavonoids, caffeine, and GC was positively correlated with the protein expression of ALDH2 in mouse liver, and negatively correlated with CYP2E1. Among the inflammatory and oxidative stress-related factors, the content of tea polyphenols, free amino acid, soluble sugar, gallic acid, EGC, C, EC, EGCG, GCG, ECG, and CG was positively correlated with the expression of TNF-α, IL-6, NRF2, and iNOS, and negatively correlated with the expression of IL-10 and HO-1.

## Discussion

AAI occurs when excessive alcohol causes the central nervous system to switch from excitement to inhibition, clinically manifested as unresponsiveness and lethargy (23). In severe cases, it leads to coma, low blood pressure, difficulty breathing, and even death (24). This study explored WEATs prepared from six types of tea on the drunken time (the time when the righting reflex losing) and the sleep time (the time when the righting reflex appeared) in AAI mice, showing that black tea and dark tea significantly shortened the sleep time and had a sobering effect. Green tea and oolong tea not only prolonged the drunken time but also shortened the sleep time and had the dual effects of slowing down the absorption and accelerating the metabolism of ethanol. White tea had the most significant effect on prolonging the drunken time, with no obvious sobering effect. Yellow tea did not affect prolonging the drunken time and shortening the sober time. There was no correlation between the drunken time and sleep time and the WEATs biochemical components. The effect of WEATs on the drunkenness and sleep of AAI mice may be the result of the combined effects of various biochemical components.

**FIGURE 10:**
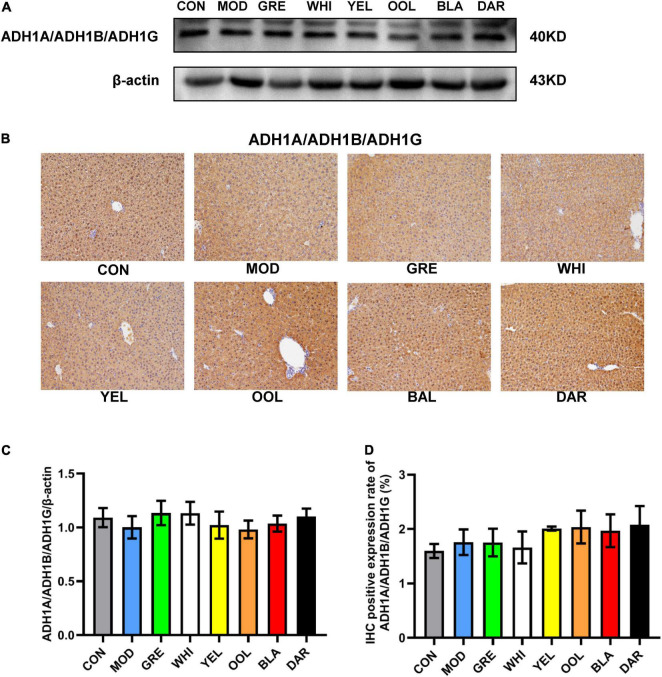
Effects of WEATs on ADH1A/ADH1B/ADH1G expression in the liver of mice. Representative immunoblots **(A)** and IHC images **(B)** of ADH1A/ADH1B/ADH1G expression in the liver. Quantification of ADH1A/ADH1B/ADH1G expression by western blotting **(C)** and IHC **(D)**. Each value represents the mean ± SEM (*n* = 10).

The liver is an important organ for ethanol metabolism, metabolizing approximately 90% of the ethanol mainly *via* oxidation by the ADH metabolic system and the microsomal ethanol oxidation system (25). ADH and ALDH are key enzymes of ethanol metabolism in the liver, which together constitute the ADH oxidative metabolism system (26). Pu-erh tea extract was shown to enhance the liver ADH and ALDH activities of chronic alcohol-exposed mice by regulating microbial metabolism (27). Another study showed that green tea significantly increased the activity of ADH in the liver of AAI mice, while ice black tea increased the activity of ALDH (28). Our results showed that black tea and dark tea significantly increased the activity of ADH and ALDH in the liver and serum, and upregulated the expression level of ALDH2 in the liver. Oolong tea could also increase the activity of ALDH both in the liver and serum and up regulate the expression of ALDH2 in the liver. However, the six WEATs tested had no effect on the expression of ADH1A/ADH1B/ADH1G protein in the liver (Figure 10). Cytochrome P450 2E1 (CYP2E1) is the main enzyme that metabolizes alcohol in the microsomal ethanol oxidation system (29). Green tea extract has been shown to downregulate the expression of CYP2E1 protein in the liver with alcoholic injury (30) but EGCG, the main catechin in green tea, had no effect on the expression of CYP2E1 in the liver induced by alcohol (31). Our results showed that black tea, dark tea and oolong tea significantly downregulated the expression of CYP2E1 protein. The fermented teas (black tea and dark tea) and semi-fermented tea (oolong tea) promoted the metabolism of ethanol by regulating the activity of serum and liver ADH and ALDH as well as the expression of ALDH2 and CYP2E1 proteins in the liver. Other low-fermented teas, especially green tea, had little or no effect on the activities of ADH, ALDH, and CYP2E1. The heat map results reflected that the tea polyphenols and catechin monomers were highly negatively correlated with ADH and ALDH activity and protein expression, and positively correlated with CYP2E1 expression, suggesting that the metabolism of ethanol by fermented teas and semi-fermented teas may be due to their polyphenol oxidation products.

AAI causes injury to digestion, nerves, and immune systems, among which the liver is the most important organ involved (32). The WEATs effectively improved liver injury caused by AAI, with liver injury scores significantly lower than those of the model group. Among them, the black tea group and the dark tea group had the lowest injury scores. The mechanisms of AAI causing liver injury are complex, including damage caused by large amounts of ethanol and its metabolite acetaldehyde, oxidative stress, and inflammation (33–35). Studies have confirmed that all six types of tea can increase the levels of SOD, GSH-Px, and GSH in the liver, and have antioxidant and hepatoprotective activities against alcohol-induced liver injury. Among them, black tea, fuzhuan brick tea, pu-erh tea, and qingzhuan brick tea have the strongest antioxidant and hepatoprotective activities in mice, while black tea has stronger antioxidant activity *in vivo* than green tea, which is contrary to the results of *in vitro* experiments (36–38). Our results show that different WEATs have different effects on oxidative stress-related indicators in the liver. Green tea and white tea alleviated the oxidative injury of the liver caused by AAI by up regulating the expression of Nrf2 and HO-1 protein and increasing the activity of GSH and CAT. Black tea, dark tea, and oolong tea slowed down the oxidative stress by increasing the activity of SOD and GSH. A previous study reported that green tea extract could regulate related inflammatory factors through the PI3K/AKT/eNOS pathway to alleviate alcoholic liver injury. In our study, the WEATs have different effects on the regulation of inflammatory factors in liver injury. Dark tea alleviated alcoholic liver injury by down-regulating TNF-α, iNOS, IL-6 and up regulating the expression of IL-10, while black tea down regulated iNOS and up regulated the expression of IL-10. Oolong tea down regulated IL-6 and up regulated the expression of IL-10 to alleviate alcoholic liver injury, while white tea down regulated TNF-α and IL-6 and up regulated IL-10. The correlation analysis also showed that the content of tea polyphenols and catechin monomers positively correlated with the expression of the pro-inflammatory factors TNF-α, iNOS, and IL-6, and negatively correlated with the expression of the anti-inflammatory factor IL-10. This suggests that the regulation of inflammation by fermented and semi-fermented teas may be due to polyphenol oxidation products and that the lack of the effect of green tea may be due to the higher content of tea polyphenols and catechins. The differences in the protective effects and mechanisms of different teas on liver injury caused by AAI need to be further studied.

## Conclusion

In summary, black tea and dark tea have a sobering effect and their mechanism of action is mainly through the regulation of key enzyme activities of ethanol metabolism, oxidative stress indexes, and inflammatory factors related to liver injury. Green tea and oolong tea have the dual effects of prolonging drunken time and shortening sleep time. The main mechanism of green tea is to regulate oxidative stress, with black tea and dark tea having similar effects to oolong tea. The effect of white tea on prolonging drunken time was the most significant among the six types of tea tested. Its mechanism may be to increase the first metabolism of ethanol in the stomach and slow down the speed of ethanol entering the blood, however, further research is required to confirm the mechanism.

## Data Availability Statement

The original contributions presented in the study are included in the article/supplementary material, further inquiries can be directed to the corresponding author/s.

## Ethics Statement

The animal study was reviewed and approved by the Ethical Committee of the Tea Research Institute (2016-002), Guangdong Academy of Agricultural Sciences.

## Author Contributions

SS and XHL: conceptualization and writing—review and editing. XFL: data curation, methodology, project administration, visualization, and writing—original draft. XFL and XW: formal analysis. LS, RC, QL, SS, and XHL: funding acquisition. XFL, XW, SW, RC, and ZZ: investigation. LS, QL, JC, ZXL, ZGL, and XHL: resources. SS: supervision. XW: validation. All authors contributed to the article and approved the submitted version.

## Conflict of Interest

The authors declare that the research was conducted in the absence of any commercial or financial relationships that could be construed as a potential conflict of interest.

## Publisher’s Note

All claims expressed in this article are solely those of the authors and do not necessarily represent those of their affiliated organizations, or those of the publisher, the editors and the reviewers. Any product that may be evaluated in this article, or claim that may be made by its manufacturer, is not guaranteed or endorsed by the publisher.
